# Rutaecarpine induces the differentiation of triple-negative breast cancer cells through inhibiting fumarate hydratase

**DOI:** 10.1186/s12967-023-04396-w

**Published:** 2023-08-18

**Authors:** Jie Lei, Yujia Pan, Rui Gao, Bin He, Zifeng Wang, Xinxing Lei, Zijian Zhang, Na Yang, Min Yan

**Affiliations:** 1 State Key Laboratory of Oncology in South China, Cancer Center, Collaborative Innovation Center of Cancer Medicine, Sun Yat-Sen University, Guangzhou, 510060 China; 2 Institute of Cancer Stem Cell, Cancer Center, Dalian Medical University, Dalian, 116023 China; 3 Department of Medical Oncology, The Seventh Affiliated Hospital, Sun Yat-Sen University, Shenzhen, 510275 China; 4 Department of Laboratory Medicine, Guangzhou First People’s Hospital, South China University of Technology, Guangzhou, 510180 China

**Keywords:** Rutaecarpine, Differentiation therapy, 3D morphological screening

## Abstract

**Background:**

Triple-negative breast cancer (TNBC) is one of the most aggressive human cancers and has poor prognosis. Approximately 80% of TNBC cases belong to the molecular basal-like subtype, which can be exploited therapeutically by inducing differentiation. However, the strategies for inducing the differentiation of TNBC remain underexplored.

**Methods:**

A three-dimensional (3D) morphological screening model based on a natural compound library was used to identify possible candidate compounds that can induce TNBC cell differentiation. The efficacy of rutaecarpine was verified using assays: RT-qPCR, RNA-seq, flow cytometry, immunofluorescence, SCENITH and label-free LC–MS/MS. The direct targets of rutaecarpine were identified through drug affinity responsive target stability (DARTS) assay. A xenograft mice model was also constructed to confirm the effect of rutaecarpine in vivo*.*

**Results:**

We identified that rutaecarpine, an indolopyridoquinazolinone, induces luminal differentiation of basal TNBC cells in both 3D spheroids and in vivo mice models. Mechanistically, rutaecarpine treatment leads to global metabolic stress and elevated ROS in 3D cultured TNBC cells. Moreover, NAC, a scavenger of ROS, impedes rutaecarpine-induced differentiation of TNBC cells in 3D culture. Finally, we identified fumarate hydratase (FH) as the direct interacting target of rutaecarpine. The inhibition of FH and the knockdown of FH consistently induced the differentiation of TNBC cells in 3D culture.

**Conclusions:**

Our results provide a platform for differentiation therapy drug discovery using 3D culture models and identify rutaecarpine as a potential compound for TNBC treatment.

**Supplementary Information:**

The online version contains supplementary material available at 10.1186/s12967-023-04396-w.

## Background

Poor differentiation is an important hallmark of cancer. Acquiring phenotype plasticity to evade or escape terminal differentiation is a critical component of cancer pathogenesis [1]. Differentiation therapy aims to reactivate endogenous differentiation programs of cancer cells to resume the maturation process, thereby altering their malignant phenotype. Ultimately, this therapy aims to alleviate the tumor burden or cure the malignant disease without damaging normal cells [2, 3]. This therapeutic approach has been successful in the treatment of acute promyelocytic leukemia (APL), a disease that is now highly curable with a combination of tretinoin (RA) and arsenic [4, 5]. Over the years, the mechanisms of cellular differentiation have been extensively explored including the genetic, epigenetic, and metabolic regulations [6–10]. However, differentiation therapy has shown limited success in other malignancies, particularly solid tumors [2].

Triple-negative breast cancers (TNBCs) are tumors that lack expression of the estrogen receptor (ER), progesterone receptor (PR), and HER2, and account for approximately 15–20% of all diagnosed breast tumors [11]. Most TNBCs are high-grade and exhibit clinically aggressive behavior. Patients with TNBCs have poor prognosis and a high risk of metastasis and death within 5 years after diagnosis [12]. Alarmingly, TNBCs lack validated therapeutic targets, and women with TNBCs do not benefit from endocrine therapy or trastuzumab. Consequently, the treatment options for TNBCs are limited, with systemic chemotherapy the current mainstay of treatment. Thus novel therapies need to be explored and developed [13]. Although TNBCs are heterogeneous, approximately 80% of TNBCs are basal-like breast cancers, which are clinically more aggressive and poorly differentiated [14, 15], providing a potential application for differentiation therapy.

Basal-like TNBCs express high levels of basal markers cytokeratin 5/14 and low levels of luminal markers cytokeratin 8/18. TNBCs also display a phenotype of epithelial-to-mesenchymal transition (EMT) and stem-like properties [16]. Interestingly, the transcription factor GATA3 has been found to specify and maintain luminal epithelial cell differentiation in the mammary gland, suppressing tumor growth and metastasis [16]. Low expression of GATA3 is associated with basal-like features and poor prognosis in breast cancers. The loss of the GATA3 function coincides with the loss of differentiation and induces basal-like mammary tumors [17, 18]. Therefore, the identification of drugs that can increase GATA3 expression and promote a luminal-like state represent possible new therapeutic strategies.

The purpose of this study was to establish a screening platform for identifying potential compounds that can effectively induce differentiation in TNBC. Three-dimensional (3D) cell culture systems have greater similarity in morphological and functional features to their original tissues and represent the minimum unit of the differentiated tissue in vivo, and therefore, provide more accurate predictions for therapeutic responses [19, 20]. Natural products usually have lower toxicity and offer more translational advantages compared to other induction agents such as transcription factor regulators [21]. In this study, we developed a miniaturized 3D cell-culture system for morphological screening and identified the natural product rutaecarpine as a candidate. In vitro 3D cell culture and an in vivo xenograft mice model revealed that rutaecarpine potently induces luminal differentiation of basal-like TNBC cells. In addition, RNA-seq and label-free LC–MS/MS assays showed that rutaecarpine induces global metabolic stress and elevated levels of reactive oxygen species (ROS) in 3D cultured TNBCs. Moreover, through a small-molecule target identification strategy termed DARTS [22], fumarate hydratase (FH) was identified as a novel binding protein of rutaecarpine. The inhibition of FH resulted in the induction of phenocopy features of rutaecarpine treatment. These data not only support the use of 3D morphological screening for differentiation therapy drug discovery but may also offer an innovative pharmacological treatment that promotes luminal differentiation in basal-like TNBCs.

## Methods

### Vector construction

To generate a lentiviral vector expressing the ATP/ADP reporter, PercevalHR was obtained from the GW1-PercevalHR (Addgene, #49082) and inserted into the pLVX-IRES-Hyg backbone using homologous recombination. The SoNar biosensor was cloned into the pLVX-IRES-Puro backbone using homologous recombination. H2B was fused to EGFP (H2B-EGFP) and cloned into the pLenti6/v5 lentiviral vector. Two pairs of shRNA for FH were cloned into the Tet-pLKO-puro (AgeI & EcoRI for sh cloning). The sequences of the shRNA pairs were: shFH-1, 5ʹ-CCGGCGCTGAAGTAAACCAGGATTACTCGAGTAATCCTGGTTTACTTCAGCGTTTTTG-3ʹ (forward) and 5ʹ-AATTCAAAAACGCTGAAGTAAACCAGGATTACTCGAGTAATCCTGGTTTACTTCAGCG-3ʹ (reverse); shFH-2, 5ʹ-CCGGGTGGTTATGTTCAACAAGTAACTCGAGTTACTTGTTGAACATAACCACTTTTTG-3ʹ (forward) and 5ʹ-AATTCAAAAAGTGGTTATGTTCAACAAGTAACTCGAGTTACTTGTTGAACATAACCAC-3ʹ (reverse).

### 3D culture

MD-MBA-231 and BT549 were plated on the surface of the Matrigel and cultured in Dulbecco’s modified Eagle medium (DMEM, GIBCO) supplemented with 10% (v/v) fetal bovine serum (GIBCO). After 6 days of culture, the gels were fixed in formalin, and subjected to immunofluorescence staining, or the gels were dissolved by cell recovery buffer for further cell experiments.

### Cell culture, transfection, and transduction

MD-MBA-231, BT549, 4T1, and HEK293 cell lines were cultured in DMEM (GIBCO) supplemented with 10% (v/v) fetal bovine serum (GIBCO). All cell lines used in this study were validated as mycoplasma free. Transfection was performed using a Lipofectamine 2000 (Invitrogen) according to the manufacturer’s instructions. To establish stable gene expression cell lines, lentivirus production was performed following our previously established procedure [23]. Cells were transduced with viral suspensions in the presence of 8 mg/ml Polybrene (Sigma-Aldrich, sc-134220) in 12-well plates. After 12 h, the lentivirus solution was replaced with fresh DMEM plus 10% FBS and seeded into 6 cm dishes and allowed to reach confluency over 48 h. Western-blotting was used to measure the shRNA interference efficiency [24]. Cells stably expressing PercevalHR, SoNar or H2B-EGFP were confirmed by confocal microscopy and purified by fluorescent cell sorting. Cells stably expressing Tet-pLKO-shFH were selected using puromycin.

### Natural product library 3D morphological screening

MD-MBA-231 cells were plated on the surface of the Matrigel and cultured for 2 days and treated with a natural drug library (Selleckchem; L1400, 10 mM DMSO stock) for 4 days in 96 well plates. DMSO was used as a vehicle control. The circularity and diameter of 3D spheroids were analyzed and quantitated using ImageJ Fiji plugins and features.

### Quantitative real-time PCR (RT-qPCR)

Total RNA was extracted using HiPure Total RNA Kits (Magen), which was used to generate cDNA by using One-Step RT-PCR SuperMix (TransScript). Quantitative RT-PCR was performed using ChamQ SYBR qPCR Master Mix (Vazyme) according to the manufacturer’s instructions. The primers used are listed in Additional file 2: Table S1. ACTB was used as the internal control.

### RNA sequencing

RNA was extracted with a HiPure Total RNA Plus Mini Kit (Magen). Library construction and RNA sequencing were constructed by Novogene with an Illumina HiSeq2000 (150 bp, paired-end). The sequencing data were qualified by fastqc (https://www.bioinformatics.babraham.ac.uk/projects/fastqc/) and the differentially expressed genes (DEG) called using the RNA Cocktail framework [25].

### Proteomic analysis by LC–MS

The MS/MS data were analyzed for protein identification and quantification using Proteome Discoverer. The false discovery rate (FDR) was 1.0% after searching against the Homo sapiens protein database, with a maximum of two missed cleavages and one missed termini cleavage (semitryptic digest). The following settings were selected: Oxidation (M), Acetylation (Protein N-term), and Deamidation (NQ), for variable modifications as well as fixed carbamidomethylation of cysteine. Precursor and fragment mass tolerance were set to 10 ppm and 0.05 Da, respectively.

### Metabolic profiling by LC–MS

The samples were thawed on ice and then 500 uL of pre-cooled extractant (80% methanol aqueous solution) was added and the mixture whirled for 2 min. Next, the ice was removed, the mixture was frozen in liquid nitrogen for five minutes and whirled again for a further two minutes. This process was carried out three times. Following that, the mixture was then centrifuged at 15,000 r/min at 4 °C for 20 min. Finally, the supernatant was poured into the sample bottle for LC–MS/MS analysis.

The sample extracts were analyzed using an LC–ESI–MS/MS system (UPLC, Shim-pack UFLC SHIMADZU CBM30A system, https://www.shimadzu.com/; MS, QTRAP^®^ System, https://sciex.com/).

### DARTS

Lysates from MDA-MB-231 cells in 2D and 3D cultures were incubated with DMSO or rutaecarpine for 1.5 h at room temperature. Lysates were then divided into certain portions and subjected to digestion with different concentrations of pronase (1074330001, Sigma Aldrich) for 20 min at room temperature. After that, the samples were boiled immediately after adding a loading buffer to stop the digestion process. The samples were then tested using SDS-PAGE and western-blotting.

### Cell cycle analysis

The cell cycle stages of cells in the 3D culture were determined by PI staining and flow cytometry. Harvested cells were adjusted to 1 × 10^6^ cells/mL and washed in cold PBS. Cells were then re-suspended and fixed in 70% cold ethanol at 4 °C overnight. One uL PI of stock solution in 1 mL of cell suspension was placed on ice for 30 min for staining. The PC5.5 channel of the CytoFLEX Platform (Beckman Coulter) was used to detect DNA content.

### Colony formation assay

For MD-MBA-231, 500 cells were plated in each well of six-well plates and treated with DMSO or rutaecarpine. The medium was renewed every third day. After 1 week, the cells were fixed using 4% paraformaldehyde for 15 min and stained with 1% crystal violet for 20 min at room temperature. After that, the crystal violet was removed and the plates washed with water several times. The plates were photographed using the ChemiDoc MP Imaging System (Bio-Rad).

### Cell migration assay

For MDA-MB-231, 1 × 10^6^ cells were plated in each well of six-well plates to create a confluent monolayer. The cell monolayer was then scraped in a straight line to create a “scratch” and capture the images for time 0. The cells were incubated with DMSO or rutaecarpine for 24 h. Following incubation, the images were captured for time 24 h. By comparing the images from time 0 to time 24 h, the distance of each scratch closure was obtained based on measurements taken by software.

### PercevalHR-based living cell ATP/ADP measurement

The PercevalHR ATP/ADP level detection was performed using flow cytometry. Three dimensional cultured cells were harvested and re-suspended in HBSS. The ratios of the FITC and KO525 channels on the CytoFLEX Platform (Beckman Coulter) were used to detect ATP/ADP levels. PH-correction was performed using a BCECF-AM pH probe (DOJINDO, B262) according to the protocol provided by the manufacturer.

### SoNar-based living cell NAD^+^/NADH measurement

The detection of NAD^+^/NADH levels was performed using flow cytometry. In brief, 3D cultured cells were harvested and re-suspended in HBSS. Ratios of F488/F405 on the CytoFLEX Platform (Beckman Coulter) were used to detect the NAD^+^/NADH level. The detailed procedures were performed as previously described [26].

### Flow cytometry analysis of ROS production

To measure the relative levels of mitochondrial superoxide, cells were re-suspended in HBSS and stained with 10 uM AM (Nanjing KeyGen Biotech., KGAF018) for 10 min at 37 °C. Cells were then washed three times with HBSS. A flow cytometer (CytoFLEX, Beckman) was used to measure ROS levels using the FITC channel.

### Extracellular acidification rate (ECAR) and oxygen consumption rate (OCR) measurements

The ECAR was measured using an Extracellular Acidification Assay kit (Abcam, ab197244) according to the manufacturer’s instructions. Briefly, the cells in the 3D culture were replaced with a 150 uL of pre-warmed culture medium per well. Then, 10 uL of reconstituted Glycolysis Assay Reagent was added. The plate was immediately read in a fluorescence plate reader over 30 min (Tecan Spark TM10M).

The extracellular OCR was measured using an Extracellular O_2_ Consumption Assay kit (Abcam, ab197243) according to the manufacturer’s instructions. Briefly, cells in the 3D culture were replaced with fresh medium containing O_2_ consumption reagent, and pre-warmed high sensitivity mineral oil was applied for air isolation. The plate was immediately read in a fluorescence plate reader over 30 min (Tecan Spark TM10M).

### SCENITH assay

The MDA-MB-231 cells in the spheroids were incubated with fresh medium for 2 h at 37 °C, 5% CO_2_ followed by treatment for 30 min with DMSO, 2-DG (40 mM; Sigma-Aldrich), oligomycin (10 uM; MCE), or a combination of both drugs. OPP reagent (20 uM; Click Chemistry Tools) was added for 20 min at 37 °C. After being washed with pre-cold PBS and collected with cell recovery buffer, cells were then fixed and permeabilized using fixation/permeabilization kit (BD Biosciences). Intracellular staining of OPP was performed with Click-&-Go^®^ Plus 555 OPP Protein Synthesis Assay Kit (Click Chemistry Tools, Catalog#1494). The intensity of intracellular OPP was quantified using the PE channel. This protocol was adapted from the original SCENITH kit (http://www.scenith.com) and the protocols developed by R. Argüello (CIML).

### FH enzyme activity assay

The MDA-MB-231 cells in the 3D culture were treated with Fumarase Assay Buffer from a Fumarase Activity Colorimetric Assay Kit (Abcam, ab196992). The cells were treated with either DMSO or rutaecarpine for 4 days in 3D culture, and Fumarate hydratase-IN-1 (MCE, HY-100004) was used as a positive control. The Fumarase Positive Control and the NADH Standard were then added to a 96-well clear-bottom plate according to the provided protocols. The absorbance at 450 nm was measured immediately in kinetic mode for 60 min at 37 °C. The FH activity was normalized to the protein concentration.

### Apoptosis analysis

The H2B-EGFP expressing cells were treated with candidate chemicals for 4 days. One uL PI of stock solution in 1 mL of PBS was used to cover the 3D spheroids and protected from light for 30 min for staining. Afterwards, all the spheroids were washed with PBS twice, and the images were taken using a Zeiss LSM 880 laser-scanning microscope.

### Mouse experiments

One × 10^5^ murine mammary carcinoma 4T1 cells in 100 uL of PBS were injected into the fourth mammary fat pad of 4- to 8-week-old BALB/c female mice. Six days after the injection, the rutaecarpine group received rutaecarpine (10 uM, 50 uL/d) via intratumoral injection, while the control group received an equal volume of PBS. The tumor volumes were calculated using the equation: *V* = 4π/3 × [(length + width)/4]^3^.

### Immunofluorescence and HE staining

The 3D spheroids were fixed with 4% paraformaldehyde for 1 h and blocked with 3% BSA at room temperature for 1 h and then incubated with Anti-KRT8 (Epitomics, 2032-1) at 1/500 dilution at 4 °C overnight. The spheroids were then incubated with Alexa Fluor 488 (Thermofisher scientific, A-11034) at a 1/300 dilution. The images were taken using a Zeiss LSM 880 laser-scanning microscope.

The tumors removed from mice were fixed with 4% paraformaldehyde overnight and embedded in OCT over liquid nitrogen. The tumors were sectioned at a thickness of 7 um, blocked with 3% BSA, and labeled with Anti-KRT18 (Abcam, ab181597) at a 1/500 dilution or Anti-Vimentin (Abcam, ab92547) at 1/500 dilution at 4 °C overnight. Primary antibodies were washed off the following day, and the cells were then incubated with anti-CD44-FITC to label cell membrane at a 1/300 dilution and Alexa Fluor 488 (Thermofisher scientific, A-11034) or Alexa Fluor 594 (Thermofisher scientific, A-11037) at a 1/300 dilution. The images were taken using a Zeiss LSM 880 laser-scanning microscope.

For Hematoxylin and Eosin (HE) Staining, tumor sections were washed with PBS for 10 min. Hematoxylin staining was performed for 3 min, followed by eosin staining for 1 min. The sections were then dehydrated with alcohol, made hyaline with xylene, and sealed. The images were taken using a NIKON ECLIPSE Ni microscope.

### Statistical analysis

Data were expressed as mean ± SD unless stated otherwise. Statistical analysis was performed using Prism GraphPad or SPSS software. Two-tailed Student’s t-test were used to compare two groups, and One-way ANOVA analysis was used to compare three or more groups. The correlation between differentiation status and spheroid circularity were analyzed by the chi-square test. For survival analysis, the Log-rank (Mantel-Cox) test was used. All *p* < 0.05 indicated a statistically significant difference. The probability values were noted as follows: * *p* < 0.05; ** *p* < 0.01; *** *p* < 0.001; **** *p* < 0.0001.

## Results

### Morphological screening of TNBC spheroids identifies that rutaecarpine promotes differentiation in 3D culture

**Fig. 1:**
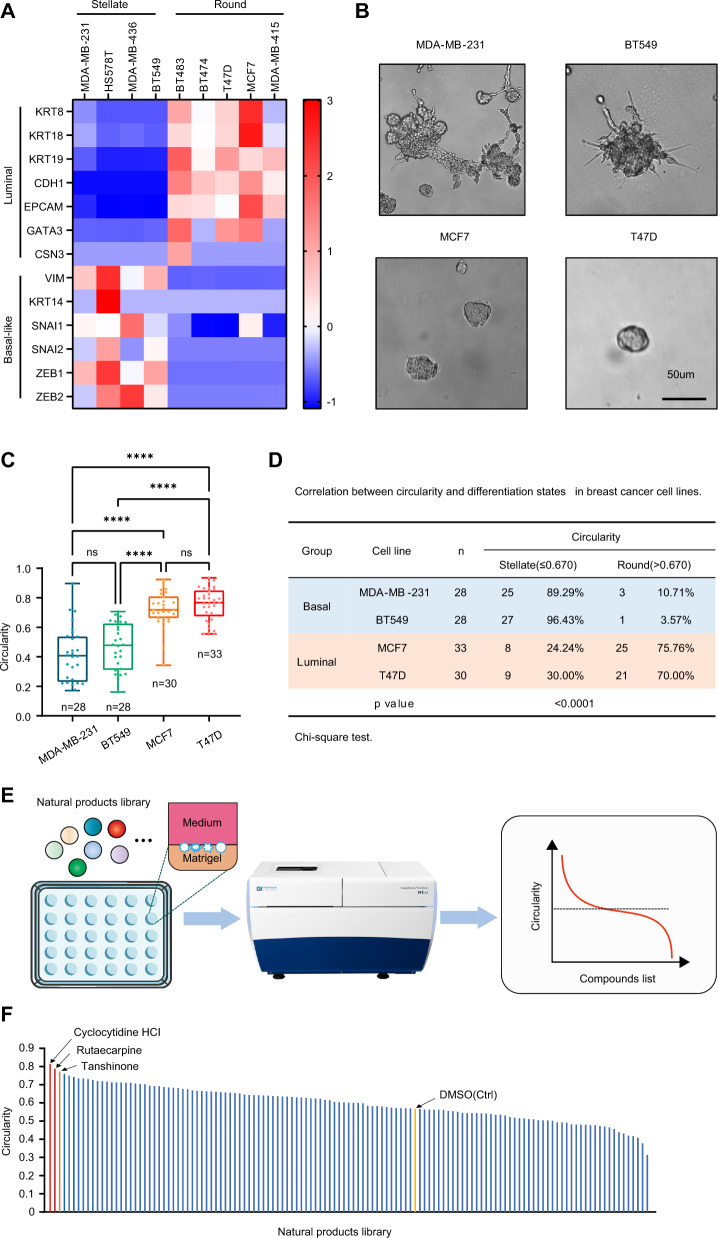
Morphological screening of TNBC spheroids identifies that rutaecarpine promotes differentiation in 3D culture. **A**. Heat map for the basal-like and luminal genes of breast cancer cell lines based on CCLE database. **B**. Spheroid formation from MDA-MB-231, BT549, MCF7 and T47D cells. **C**. Quantification of circularity for spheroids formed by MDA-MB-231, BT549, MCF7 and T47D cells. One-way ANOVA; **** *p* < 0.0001. **D**. A cutoff value that best discriminated between groups with high or low circularity with respect to differentiation status was determined using the maximal Youden’s index. Chi-square test. **E**. Schematic for morphological screening to identify natural products that promote differentiation of MDA-MB-231 cells in 3D culture. **F**. Plot showing the efficiency of natural products in regulating the circularity of MDA-MB-231 spheroids in 3D culture. The top three natural products and DMSO are highlighted

Previous studies have found a correlation between gene pattern and morphology in 3D cultured breast cancer cells [27, 28]. In contrast to luminal cell lines, TNBC basal cell lines such as MDA-MB-231, BT549, HS578T, and MDA-MB-436 form stellate spheroids with high aggressivity in 3D culture. Upon induction of luminal differentiation, these spheroids switch to a round morphology [16]. To confirm the correlation between differentiation status and morphology, we analyzed the differentiation genes of human breast cancer cell lines using TPM values from the Cancer Cell Line Encyclopedia (CCLE). Consistently, basal-like breast cancer cells form stellate spheroids, while luminal breast cancer cells form round spheroids in 3D culture (Fig. 1A and B). The circularities of luminal spheroids are significantly higher than those of basal-like spheroids (Fig. 1C). An analysis using the Chi-square test showed a positive correlation between spheroid circularity and luminal differentiation status (*p* < 0.0001, Fig. 1D). Thus, a round morphology is an indicator of luminal differentiation status.

To identify potential small molecules that promote differentiation of basal-like breast cancer, we performed 3D morphological screening with a natural product library. In brief, MDA-MB-231 cells were plated on the surface of the Matrigel, cultured for 2 days, then treated with a natural drug library (Selleckchem) comprising 144 natural products for 4 days in 96 well plates. The circularity of 3D spheroids was analyzed and quantified using ImageJ Fiji (Fig. 1E). The screening results were then sorted according to the circularity of the 3D spheroids, with round morphology at the top and stellate morphology at the bottom. Several natural products, such as cyclocytidine HCI, rutaecarpine, and tanshinone IIA were identified to induce a morphology switch from stellate to round in the 3D cultured MDA-MB-231 cells (Fig. 1Fand Additional file 1: Figure S1A). As cyclocytidine and tanshinone have been reported to suppress various cancer growth [29, 30], we chose rutaecarpine for further study.

**Fig. 2:**
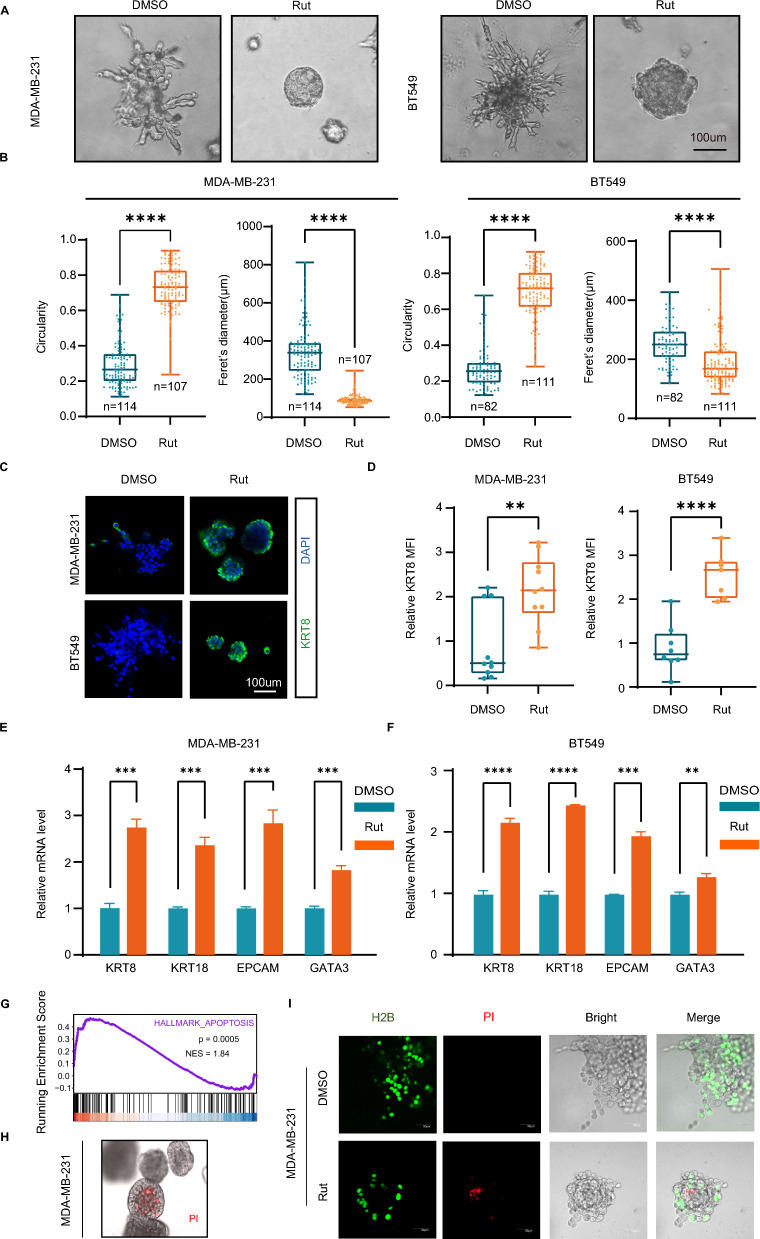
Rutaecarpine induces luminal differentiation of TNBC cells in 3D culture. **A**. Spheroid formation from MDA-MB-231 and BT549 cells with DMSO or rutaecarpine treatment. **B**. Quantification of circularity and Feret’s diameter for spheroids formed by MDA-MB-231 and BT549 cells. Unpaired Student’s t-test; **** p < 0.0001. **C**. IF staining of KRT8 in DMSO or rutaecarpine-treated MDA-MB-231 and BT549 spheroids. **D**. Quantification of KRT8 MFI of the DMSO or rutaecarpine-treated MDA-MB-231 and BT549 spheroids. Unpaired Student’s t-test; ** p < 0.01; **** p < 0.0001. **E**. qPCR of luminal marker genes in DMSO or rutaecarpine-treated MDA-MB-231 spheroids. Unpaired Student’s t-test; *** p < 0.001. **F**. qPCR of luminal marker genes in DMSO or rutaecarpine-treated BT549 spheroids. Unpaired Student’s t-test; ** p < 0.01; *** p < 0.001; **** p < 0.0001. **G**. GSEA analysis showing significant enrichment of apoptosis genes in rutaecarpine-treated MDA-MB-231 cells in 3D spheroids. **H**. Apoptosis analysis for the matured MCF-10A spheroids with PI staining. **I**. Apoptosis analysis for DMSO or rutaecarpine-treated MDA-MB-231 cells stably expressing H2B-EGFP with PI staining

To examine the function of rutaecarpine in inducing the differentiation of basal-like breast cancer, we analyzed the morphologies of MDA-MB-231 and BT549 cells with rutaecarpine treatment for 4 days in 3D culture. Consistent with the screening results, both MDA-MB-231 and BT549 formed round spheroids with a smaller diameter under the treatment of rutaecarpine (Fig. 2A and B). Immunofluorescence staining showed higher expression of the luminal gene KRT8 in rutaecarpine treated spheroids (Fig. 2C and D). In addition, rutaecarpine increased the mRNA expression of the luminal genes, such as KRT8, KRT18, EPCAM, and GATA3 in 3D cultured spheroids (Fig. 2E and F). Collectively, these results indicate that rutaecarpine promotes the basal-like TNBC cells differentiated into luminal-like cells in 3D culture.

### Rutaecarpine induces differentiation of TNBC cells through elevated ROS

Rutaecarpine has been reported to improve lung dysfunction through its classic function as a COX2 inhibitor [31, 32]. However, the COX2 inhibitor rofecoxib does not induce the differentiation of MDA-MB-231 cells in 3D culture (Additional file 1: Figure S1B). Since rutaecarpine-treated 3D spheroids have a smaller diameter, we then explored whether rutaecarpine induces the differentiation of TNBC cells by limiting the proliferation or migration of cancer cells. Unexpectedly, rutaecarpine treatment neither induces cell cycle arrest, reduces colony formation, nor limits cell migration in 2D cultured MDA-MB-231 cells (Additional file 1: Figure S2A, B, C and D).

**Fig. 3:**
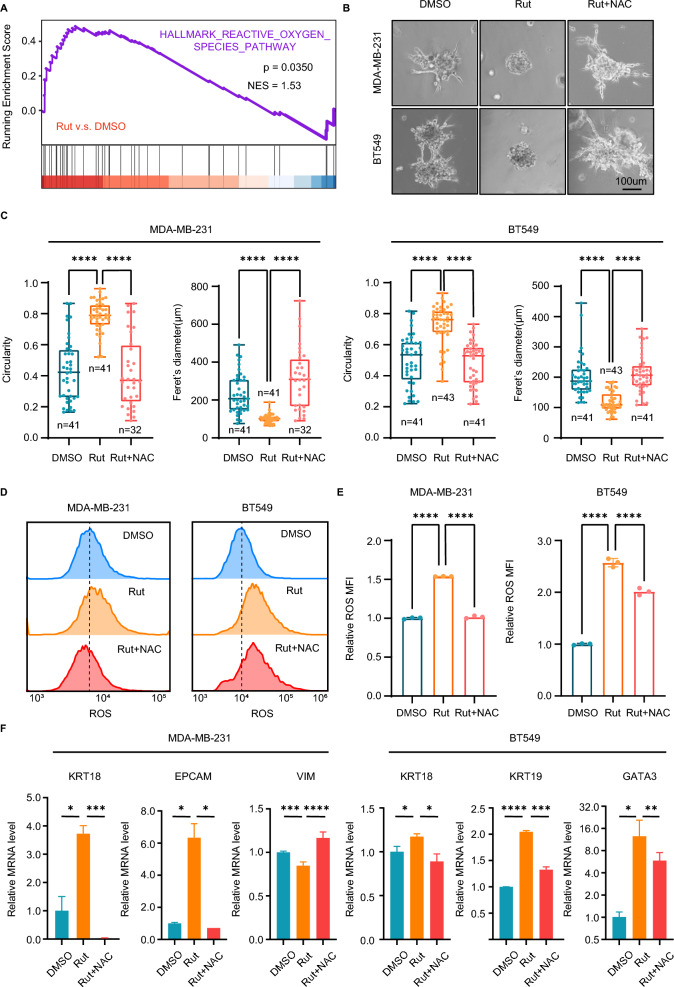
Rutaecarpine induces differentiation of TNBC cells through elevated ROS. **A**. GSEA analysis showing significant enrichment of ROS genes. **B**. Spheroid formation from MDA-MB-231 and BT549 cells with the treatment of DMSO or rutaecarpine and NAC. **C**. Quantification of circularity and Feret’s diameter for spheroids formed by MDA-MB-231 and BT549 cells. One-way ANOVA; **** *p* < 0.0001. **D**. FCM analysis of the MFI of ROS in cells from spheroids. **E**. Quantification of ROS level in cells from spheroids. One-way ANOVA; **** *p* < 0.0001. **F**. qPCR of luminal differentiation genes in cells from MDA-MB-231 and BT549 spheroids with different treatment. One-way ANOVA; * *p* < 0.05; ** *p* < 0.01; *** *p* < 0.001; **** *p* < 0.0001

To explore the mechanism of rutaecarpine in promoting TNBC cell differentiation, we performed RNA-sequencing (RNA-seq) on MDA-MB-231 cells and rutaecarpine-treated MDA-MB-231 cells purified from 3D organoids. The gene set enrichment analysis (GSEA) displayed enrichment in the ROS pathway (NES = 1.53, *p* < 0.05) as well as the apoptosis pathway (NES = 1.84, *p* < 0.001) (Fig. 2G and 3A). Consistent with the results of the GSEA, PI staining also showed that rutaecarpine increases the apoptosis of MDA-MB-231 cells in the central part of the 3D spheroids, which represents the maturation of luminal structures similar to the differentiation process of MCF-10A cells in 3D spheroids (Fig. 2H and I).

Several articles have reported that ROS dynamics control the differentiation states of neuron progenitor cells or breast cancer cells [33, 34]. To investigate whether rutaecarpine induces the differentiation of TNBC cells through increasing ROS, we treated the rutaecarpine-induced differentiated 3D spheroids with NAC, the scavenger of ROS. Expectedly, NAC reverses the elevated ROS level, spheroid morphologies, and molecular features caused by rutaecarpine treatment (Fig. 3B, C, D, E and F). Taken together, these results indicate that rutaecarpine induces the differentiation of TNBC cells in 3D culture through increasing ROS level.

### Rutaecarpine induces metabolic reprogramming in TNBC cells

**Fig. 4:**
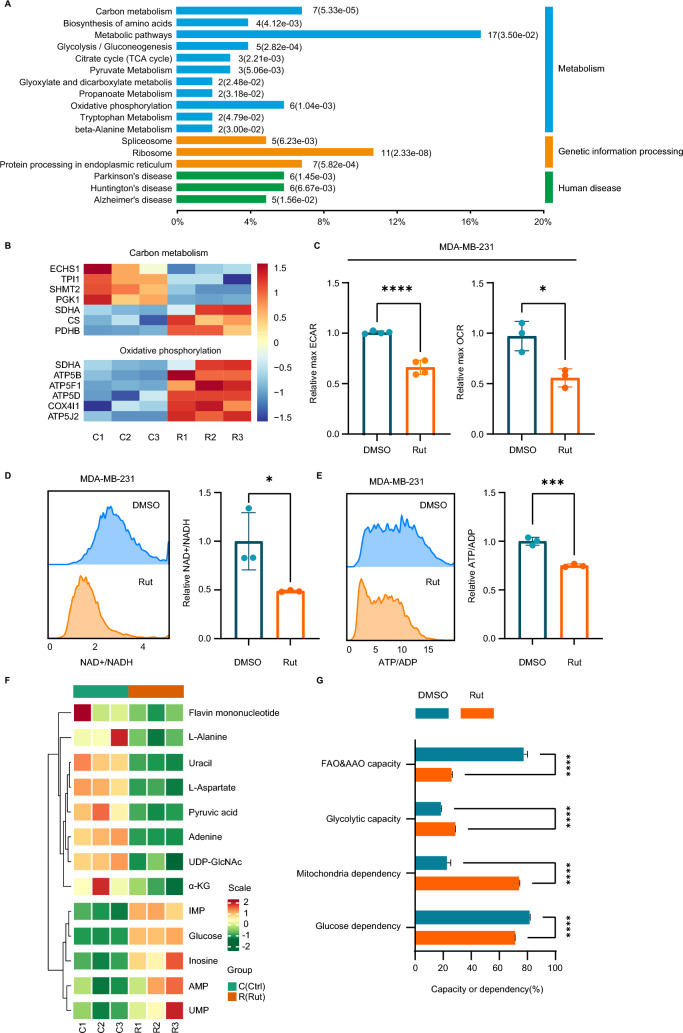
Rutaecarpine induces metabolic reprogramming in TNBC cells. **A**. KEGG analysis showing significant enrichment in metabolism, genetic information processing and human disease. **B**. Heat map of carbon metabolism and oxidative phosphorylation. **C**. ECAR and OCR test with TECAN for 1.5 h for the cells in 3D spheroids and quantification. Unpaired Student’s t-test; * p < 0.05; **** p < 0.0001. **D**. FCM test of SoNar-expressing cells in spheroids. Quantification of intracellular F488/F405 (NAD.^+^/NADH) ratios in single cells resuspended in HBSS buffer. Unpaired Student’s t-test; * p < 0.05. **E**. FCM analysis of PercevalHR-expressing cells in spheroids. Quantification of intracellular F488/F405 (ATP/ADP) ratios in single cells resuspended in HBSS buffer. Unpaired Student’s t-test; *** p < 0.001. **F**. Heat map of metabolomic analysis of DMSO- and rutaecarpine-treated MDA-MB-231 cells in spheroids. **G**. SCENITH test of the MDA-MB-231 cells in spheroids DMSO or rutaecarpine treatment. Student’s t-test; **** p < 0.0001

ROS are byproducts of biological reactions of energy generation and are primarily produced in the mitochondria through oxidative metabolism [35]. To explore how rutaecarpine induces elevated ROS of TNBC cells in 3D spheroids, we performed proteomic analysis on MDA-MB-231 cells and rutaecarpine-treated MDA-MB-231 cells purified from 3D spheroids. Kyoto Encyclopedia of Genes and Genomes (KEGG) pathway analysis of 92 differentially expressed proteins revealed significant enrichment in the metabolic pathways, especially in carbon metabolism and oxidative phosphorylation (Fig. 4A and B).

Previous studies have shown that metabolic stress could induce elevated ROS and limit cancer invasiveness through inducing the differentiation of cancer cells, especially in glioblastoma [10, 36–38]. We then asked whether rutaecarpine induces elevated ROS as a result of metabolic stress or raised oxidative phosphorylation. Firstly, we assessed the ECAR and OCR of MDA-MB-231 cells in 3D spheroids and found that MDA-MB-231 cells have lower ECAR and OCR under the induction of rutaecarpine, denoting a reduced glycolytic rate and oxidative phosphorylation (Fig. 4C). These results suggest that the elevated ROS may not be due to the increased electron transport chain (ETC) flux (Fig. 4B and C).

To further understand the metabolic states of the cells, we quantified the ratio of ATP/ADP using the fluorescent reporter PercevalHR [39] and the ratio of NAD^+^/NADH with the fluorescent reporter SoNar [26] at the single cell dimension. In brief, MDA-MB-231 cells expressing the reporters were seeded on a Matrigel substrate. After spheroid maturation, the cells were collected with a cell recovery buffer, and the ratios monitored in real-time using flow cytometry. Consistent with the results of ECAR and OCR, rutaecarpine-treated MDA-MB-231 cells entered a metabolic quiescent state with a lower ratio of ATP/ADP and NAD^+^/NADH (Fig. 4D and E). Energy metabolomic analysis also showed a significant change in carbon metabolism, including the accumulation of glucose and AMP along with reduced pyruvate and a-KG (Fig. 4F). To gain an insight into the metabolic features of rutaecarpine-treated MDA-MB-231 cells, we analyzed the cells in spheroids using SCENITH assay [40]. MDA-MB-231 cells in the spheroids displayed a significantly altered metabolic profile towards mitochondrial metabolism with reduced glucose dependence following rutaecarpine treatment (Fig. 4G). These results suggest that rutaecarpine-treated cells with impaired glucose catabolism entered an advanced state of cellular starvation.

### FH is identified as the direct target of rutaecarpine

**Fig. 5:**
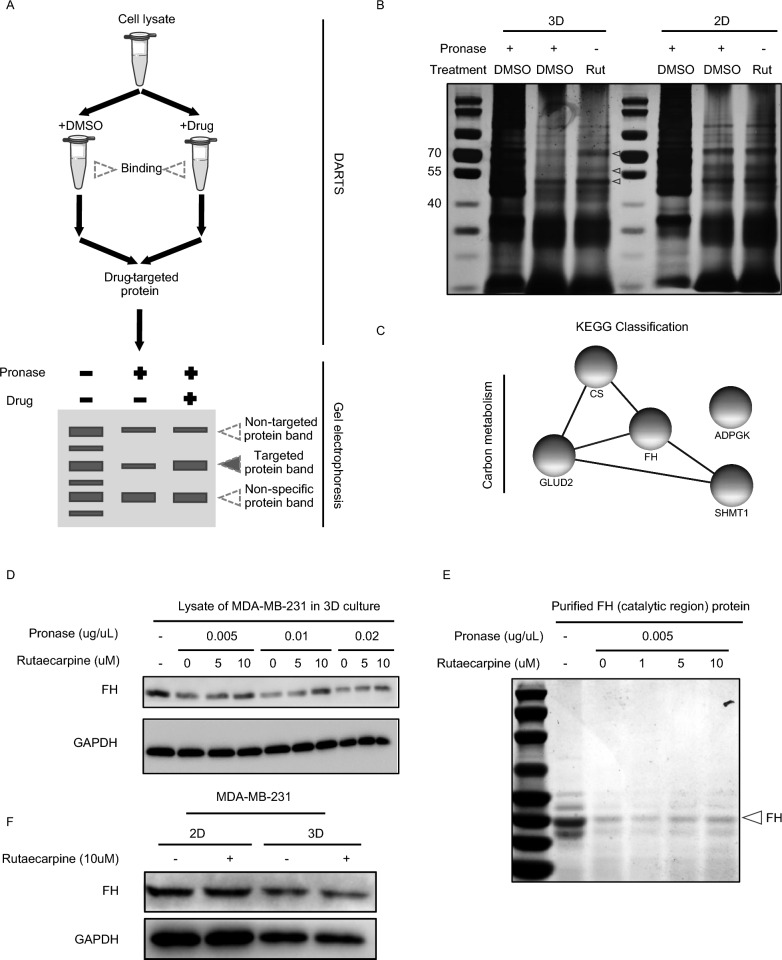
FH is identified as the direct target of rutaecarpine. **A**. Schematic for DARTS assay to identify the target of small molecules. **B**. SDS-PAGE for the DARTS assay, highlighting and purifying the potential targeting proteins specific to 3D and 2D culture for further MS analysis. **C**. KEGG analysis for the potential targeting proteins of rutaecarpine showing enrichment in carbon metabolism. **D**. Western-blotting showing a dose dependent function of rutaecarpine in preventing the degradation of FH by pronase in cell lysates from MDA-MB-231 cells in spheroids. **E**. Western-blotting showing a dose dependent function of rutaecarpine in preventing the degradation of FH purified from bacteria. **F**. Western-blotting showing that rutaecarpine has no effect in regulating the protein level of FH in MDA-MB-231 cells from 2D and 3D cultures

Given that rutaecarpine induces the differentiation of TNBC cells through elevated ROS without increased ETC flux, we hypothesized that rutaecarpine has other targets that influence the generation or elimination of ROS. To test this, we performed the DARTS assay to identify the direct target of rutaecarpine. Equal amounts of cell lysates from MDA-MB-231 cells in 2D culture or 3D culture were mixed with rutaecarpine (10 uM) for 1.5 h and then incubated with pronase for 20 min at room temperature. The samples were then loaded on SDS-PAGE gels, and the gels stained with Coomassie blue (Fig. 5A). As shown in the SDS-PAGE gel, three protein bands were increased in the rutaecarpine-treated cell lysates from the 3D organoids specifically (Fig. 5B). Mass spectrometry analysis identified several proteins involved in carbon metabolism which may be the potential target of rutaecarpine (Fig. 5C).

Given that rutaecarpine induces metabolic stress on TNBC cells mainly from impaired glucose catabolism (Fig. 4F and G), we selected CS and FH, two core components of the tricarboxylic acid (TCA) cycle for further verification (Fig. 5C). Through a secondary DARTS assay, we confirmed that FH, but not CS, is the direct target of rutaecarpine in 3D spheroids, as FH level increased in the rutaecarpine-treated cell lysates in a dose-dependent manner under pronase addition (Fig. 5D and Additional file 1: Figure S3A). Purified FH protein and additional tests also indicated that rutaecarpine can directly bind to FH (Fig. 5E). Together, these results suggest that rutaecarpine promotes the differentiation of TNBC cells by directly targeting FH.

### Inhibition of FH induces the differentiation of TNBC cells through elevated ROS

As rutaecarpine directly binds to FH, we investigated whether rutaecarpine affects the protein abundance of FH. Consistent with the results from the proteomic analysis (Fig. 4B), Western-blot results indicated that rutaecarpine does not affect FH levels in MDA-MB-231 cells in either 2D or 3D cultures (Fig. 5F).

FH is an enzyme involved in the TCA cycle and converts fumarate to malate. Although the TCA cycle is blocked by loss of FH activity, it can still operate in reverse to metabolize glutamate to citrate by reductive carboxylation [41]. Inhibiting the TCA cycle in this manner can potentially increase the generation of ROS by halting the flux of electrons at iron-sulphur centers or flavin groups. Trapped in the ‘traffic gridlock,’ these electrons may be captured by O_2_ to generate superoxide [42, 43]. Consistent with our observations, rutaecarpine-treated cells upregulate the components of mitochondrial complex V (ATP synthase) to meet the energy demand (Fig. 4B), which may be accompanied by the generation of ROS. Thus, we propose that rutaecarpine blocks the enzymatic activity of FH, leading to the induction of elevated ROS.

**Fig. 6:**
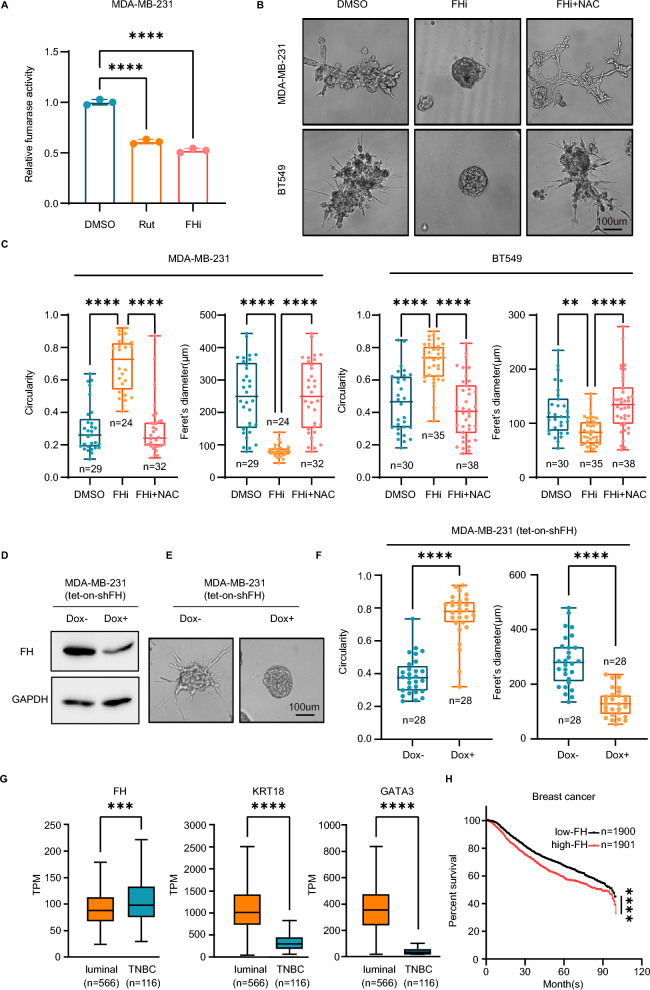
Inhibition of FH induces differentiation of TNBC cells through elevated ROS. **A**. Quantification of fumarase activity of MDA-MB-231 cell lysates under different treatments using a fumarase kit. An FH inhibitor was used as a positive control. One-way ANOVA; **** *p* < 0.0001. **B**. Spheroid formation from MDA-MB-231 and BT549 cells following treatment with DMSO, FH inhibitor, and NAC. **C**. Quantification of circularity and Feret’s diameter for spheroids formed by MDA-MB-231 and BT549 cells. One-way ANOVA; **p < 0.01; ****p < 0.0001. **D**. Western-blotting showing a reduction in FH in protein level with the knockdown of shRNA of FH in MDA-MB-231 cells. **E**. Spheroid formation from MDA-MB-231 cells with, and without the knockdown of FH. **F**. Quantification of circularity and Feret’s diameter for spheroids formed by MDA-MB-231 cells with, and without the knockdown of FH. Unpaired Student’s t-test; **** p < 0.0001. **G**.TPM analysis of FH, KRT18 and GATA3 genes in tumors from luminal and TNBC patients based on TCGA database. Unpaired Student’s t-test; *** p < 0.001; **** p < 0.0001. **H**. Relapse-free survival (RFS) classified by the FH transcription levels in breast cancer patients. Log-rank (Mantel-Cox) test; **** p < 0.0001

To test whether rutaecarpine functions as an inhibitor of FH, we treated the cell lysates of MDA-MB-231 cells with rutaecarpine and used an FH inhibitor as a positive control. Remarkably, rutaecarpine induced a blockage of FH activity similar to that of the FH inhibitor (Fig. 6A). Consistently, the FH inhibitor also induced the differentiation of TNBC cells in 3D spheroids through elevated ROS, which could be reversed by NAC treatment (Fig. 6B and C, Additional file 1: Figure S3B, C&D). Moreover, inducible knockdown of FH expression promoted the differentiation of MDA-MB-231 cells (Fig. 6D, E and F). Overall, rutaecarpine induces elevated ROS by inhibiting the activity of FH, thereby promoting the luminal differentiation of TNBC cells in 3D spheroids.

Lastly, we analyzed the prognostic values of FH and the luminal genes in breast cancer based on The Cancer Genome Atlas (TCGA) database. The expression of FH was elevated while the expressions of luminal genes, KRT18 and GATA3 were decreased in TNBC patients compare with luminal type patients (Fig. 6G). Additionally, FH expression was identified as an independent prognostic factor in breast cancer patients (Fig. 6H). Taken together, these results demonstrate that rutaecarpine induces luminal differentiation of TNBC cells in 3D spheroids through metabolic reprogramming.

### Rutaecarpine induces differentiation of TNBC in vivo

**Fig. 7:**
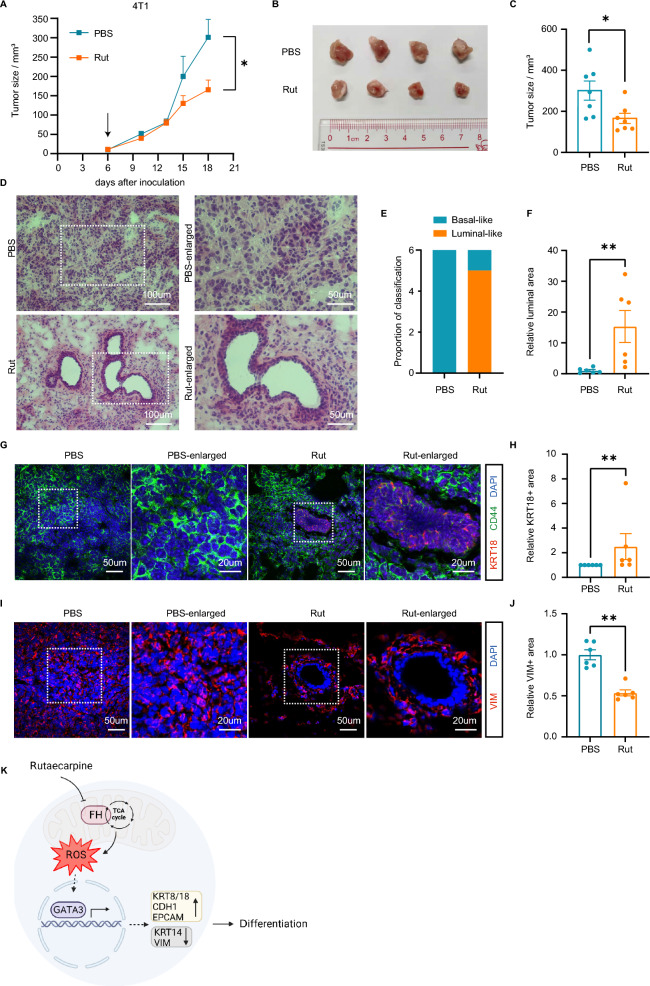
Rutaecarpine induces differentiation of TNBC in vivo. **A**. The growth curves of xenograft tumors derived from 4T1 cells. Mice were subjected to daily treatments with PBS or rutaecarpine. Unpaired Student’s t-test; * p < 0.05. Mean ± SEM. **B**. The 4T1 tumors removed from mice in each group are shown. **C**. Statistical analysis of the volume of the dissected tumors at end points. Unpaired Student’s t-test; * p < 0.05. Mean ± SEM. **D**. HE staining for the tumor slides. **E**. Proportion analysis for the tumor slides with luminal structure. **F**. Statistical analysis of the luminal-like area of the tumor slides. Unpaired Student’s t-test; ** p < 0.01. Mean ± SEM. **G**. IF staining of KRT18 and CD44 for the tumor slides. **H**. Statistical analysis of the KRT18 positive area of the tumor slides. Unpaired Student’s t-test; ** p < 0.01. Mean ± SEM. **I**. IF staining of VIM for the tumor slides. **J**. Statistical analysis of the VIM positive area of the tumor slides. Unpaired Student’s t-test; ** p < 0.01. Mean ± SEM. **K**. Working model for the mechanism of rutaecarpine induced differentiation of TNBC cells in 3D culture

To examine the efficacy of rutaecarpine in inducing differentiation in vivo, we used a xenograft mouse model with BALB/c mice bearing 4T1 xenografts. The mice were administered a vehicle or rutaecarpine via intratumoral injection. Consistent with our in vitro findings, rutaecarpine treatment resulted in a significant inhibition of tumor growth compared to the control, as evident from the decreased tumor sizes (Fig. 7A, B and C). We further examined whether the anti-tumor function of rutaecarpine comes from its differentiation induction efficacy. Notably, we found that rutaecarpine-treated 4T1 cells form luminal-like structures within the tumors and that each rutaecarpine-treated tumor has a broader luminal-like area compared to the control vehicle-treated tumors (Fig. 7D, E and F). In addition, to the morphology conversion, there was a significant change in the levels of the differentiation gene. Rutaecarpine-treated tumors had a larger KRT18 positive area and a smaller VIM positive area compared to the vehicle-treated tumors, indicating that rutaecarpine induces the luminal differentiation of 4T1 cells (Fig. 7G, H, I and J). Thus, the xenograft mouse model demonstrated strong evidence for the differentiation induction potential of rutaecarpine in vivo.

## Discussion

Breast cancer is the most frequent malignancy in women and is the second leading cause of cancer-related deaths among women worldwide [44]. While considerable progress has been made in terms of the diagnosis and treatment of non-TNBCs, which has led to more effective treatment and improved patient survival [11], TNBC patients with worse prognoses still lack target therapeutic strategies [45].

**Fig. 8:**
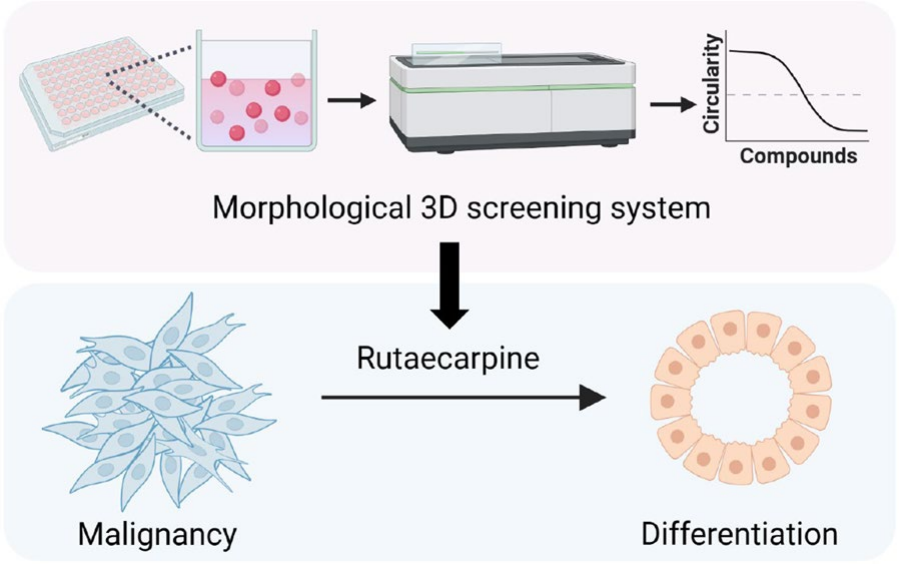
Graphic illustration of the differentiation therapy with rutaecarpine. Upper panel: Schematic for morphological screening to identify natural products that promote differentiation of TNBC cells in 3D culture. Lower panel: Rutaecarpine decreases the malignancy of TNBC cells in 3D culture through differentiation induction

Here in this study, we generated a 3D spheroid screen based on morphological features and found that rutaecarpine-induced the differentiation of TNBC cells, suppressed tumor growth, and restored a luminal-like structure in vivo. Mechanically, rutaecarpine increased cellular ROS and reduced the levels of ECAR, OCR, NAD^+^/NADH, and ATP/ADP in TNBC cells. Moreover, FH was identified as a novel binding protein of rutaecarpine using DARTS. Furthermore, the inhibition of FH increased cellular ROS and induced the differentiation of TNBC cells in 3D culture, while NAC reversed these phenotypes. These data provide compelling evidence that rutaecarpine is a potential drug for differentiation therapy in patients with basal-like TNBCs (Figs. 7K and 8).

Several previous efforts have been made to screen for differentiation targets of TNBCs. Such studies used the transcription or expression of differentiation markers such as KRT5, KRT8, or CDH1, to identify potential targets or compounds in 2D culture breast cancer cell lines [46, 47]. However, 2D culture models are unable to simulate the cell–cell interaction and tissue structure in normal breast tissue, which is an important feature of differentiation. Notably, a recent study compared 2D and 3D screening models and found that CRISPR phenotypes in 3D models more accurately reflected those of in vivo tumors, and revealed drivers that are essential for cancer growth in 3D and in vivo, but not in 2D models [48].

In this study, we developed a 3D morphological screening platform to screen for drugs that can induce the differentiation of TNBCs. In doing so, rutaecarpine was identified as an effective compound. Rutaecarpine treatment not only altered the levels of differentiation markers but also displays a round spheroid morphology in a 3D culture model that is unique to normal luminal breast cells. When applied in vivo, rutaecarpine also exhibited potent anti-tumor capabilities that induced the formation of luminal-like structures resembling normal mammary glands. This morphological screening platform therefore provides a valuable tool for differentiation therapy drug discovery.

As a natural product, rutaecarpine exhibits various activities, including anti-inflammatory, antithrombotic, and neuronal protection [31, 49, 50]. It has also been shown to have antitumor characteristics through inhibiting cell proliferation and promoting apoptosis [51]. Yet, its function in promoting tumor differentiation and the underlying molecular mechanisms have not been elucidated. In this study, we demonstrated that rutaecarpine reverses the malignant phenotype and induces the differentiation of basal-like TNBCs in 3D culture and in vivo. Rutaecarpine treatment leads to an increase in levels of luminal differentiation markers cytokeratin 8/18 as well as decreased levels of the basal marker vimentin. In 3D culture models, rutaecarpine alters the morphology of basal-like TNBC MDA-MB-231 spheroids and induces apoptosis of the cells inside the spheroids, eventually forming acini-like structures similar to those of immortalized MCF-10A cells. Moreover, in a xenograft mice model, rutaecarpine induced the formation of luminal structures that resemble normal human breast tissue. These findings suggest that rutaecarpine is a promising drug candidate for differentiation therapy of basal-like TNBCs.

Although excessive ROS induces DNA mutation and genomic instability, or as a signal molecule, accelerates tumor cell proliferation, survival, and metastasis, a moderate amount of ROS is essential for normal differentiation and development [35, 52]. For example, ROS-mediated changes in mitochondrial dynamics thereby regulate stem cell fate decisions [53, 54]. Notably, increased ROS is involved in the clearance of matrix-deprived cells, which plays a key role during mammary gland development [55, 56]. In addition, the modulation of redox signaling promotes the transition of breast cancer stem cells from mesenchymal-like to epithelial-like states [34]. Consistently, we found that rutaecarpine treatment increases cellular ROS in breast cancer cells and alters metabolic patterns. We further identified FH as the principal pharmacological target of rutaecarpine. Germline mutations in the FH gene are also correlated with an increased risk of tumorigenesis [57, 58], which indicates FH is a tumor suppressor. However, the inhibition of FH exhibits anti-proliferative activities in a variety of cancer cells [59]. We found that the FH inhibitor increases cellular ROS, and induces the differentiation of basal breast cells in 3D culture models. The antioxidant NAC also reversed the phenotypes induced by rutaecarpine treatment or FH inhibition. These results suggest that rutaecarpine induces the differentiation of basal-like TNBC cells by modulating redox signaling.

In this study, we established a 3D morphologic-functional screening model that better simulates the differentiation features of epithelial tissues compared with 2D models. Using this method, we identified rutaecarpine as a potential candidate for inducing the differentiation of TNBCs. The efficacy of rutaecarpine for inducing the differentiation of TNBCs was verified in both 3D spheroids and in vivo models. Despite these highly encouraging results, there are several questions that need to be addressed in future studies. Firstly, while we demonstrated that rutaecarpine promotes luminal differentiation in a xenograft mouse model, further investigations are required to determine the efficacy of rutaecarpine in inducing differentiation in spontaneous breast cancer mouse models and patient-derived transplanted tumor models. Secondly, our study revealed that rutaecarpine induces differentiation through metabolic modulation, yet this may only represent a fraction of the underlying mechanisms. Further investigations into other aspects such as epigenetic regulation are required for a comprehensive understanding. Finally, although the animals treated with rutaecarpine did not exhibit any reduction in body weight during our observation, more rigorous exploration is needed to assess the potential long-term toxicity of rutaecarpine.

## Conclusion

Poor differentiation is a hallmark of basal TNBCs and is associated with poor patient prognosis. In this study, we developed a morphological high-throughput screening model and identified that rutaecarpine effectively induces the differentiation of TNBCs in 3D culture and in vivo. These data provide a discovery platform to identify drugs that induce differentiation and suggest that rutaecarpine may have therapeutic potential in the differentiation therapy of TBNCs. Furthermore, dedifferentiation has emerged as a general trait of cancer evolution and a driver of resistance to immunotherapy [60, 61]. Recently, immunotherapy represents a potential treatment strategy in TNBCs [62]. Exploring the combination of rutaecarpine with immune checkpoint inhibitors may be promising.

### Supplementary Information

**Additional file 1: Figure S1.** Related to Figs. 1 and 2: Rutaecarpine, but not COX2 inhibitor, induces differentiation of TNBC cells in 3D culture. A. Spheroid formation from MDA-MB-231 cells treated with the top three candidate drugs from the 3D screening, using DMSO as a vehicle control. Cyclocytidine HCl, 10 uM; Rutaecarpine, 10 uM; Tanshinone IIA, 10 uM. B. Spheroid formation from MDA-MB-231 cells treated with DMSO, rutaecarpine, and a COX2 inhibitor, rofecoxib. **Figure S2.** Related to Fig. 2: Rutaecarpine has no effect in either cell cycle, colony formation or migration of MDA-MB-231 cells. A. Cell cycle analysis of MDA-MB-231 cells purified from spheroids treated with DMSO or rutaecarpine in 3D culture. B. Colony formation assay of MDA-MB-231 cells treated with DMSO or rutaecarpine in 2D culture. C. Quantification of the colony formation assay of MDA-MB-231 cells. Unpaired Student’s t-test; ns, *p* > 0.05. D. Quantification of the migration assay of MDA-MB-231 cells with the treatment of DMSO or rutaecarpine in 2D culture. Unpaired Student’s t-test; ns, *p* > 0.05. **Figure S3.** Related to Fig. 5: FH, but not CS, is the direct target of rutaecarpine. A. DARTS assay to identified the target of rutaecarpine in spheroids formed by MDA-MB-231 cells using western-blotting. B. FCM test for the ROS of MDA-MB-231 and BT549 spheroids treated with DMSO, FH inhibitor, or a combination of FH inhibitor and NAC. C. Quantification of ROS MFI in spheroids formed by MDA-MB-231 and BT549 spheroids. One-way ANOVA; * p < 0.05; ** p < 0.01. D. qPCR of luminal marker genes in DMSO or FH inhibitor-treated MDA-MB-231 and BT549 spheroids. Unpaired Student’s t-test; * p < 0.05; ** p < 0.01; *** p < 0.001; **** p < 0.0001.

**Additional file 2:** Primers for amplification of indicated genes.

## Data Availability

The raw data for RNA-Seq is available in the Genome Sequence Archive (Genomics, Proteomics & Bioinformatics 2017) database with the accession number HRA001209.
