# Supplementary material for: Rutaecarpine induces the differentiation of triple-negative breast cancer cells through inhibiting fumarate hydratase
Source: J Transl Med. 2023 Aug 18;21:553. doi: 10.1186/s12967-023-04396-w (PMC10436383; doi:10.1186/s12967-023-04396-w)

Extended Data Fig. 1 | Rutaecarpine does not induce differentiation of TNBC cells through inhibiting COX2 in 3D culture

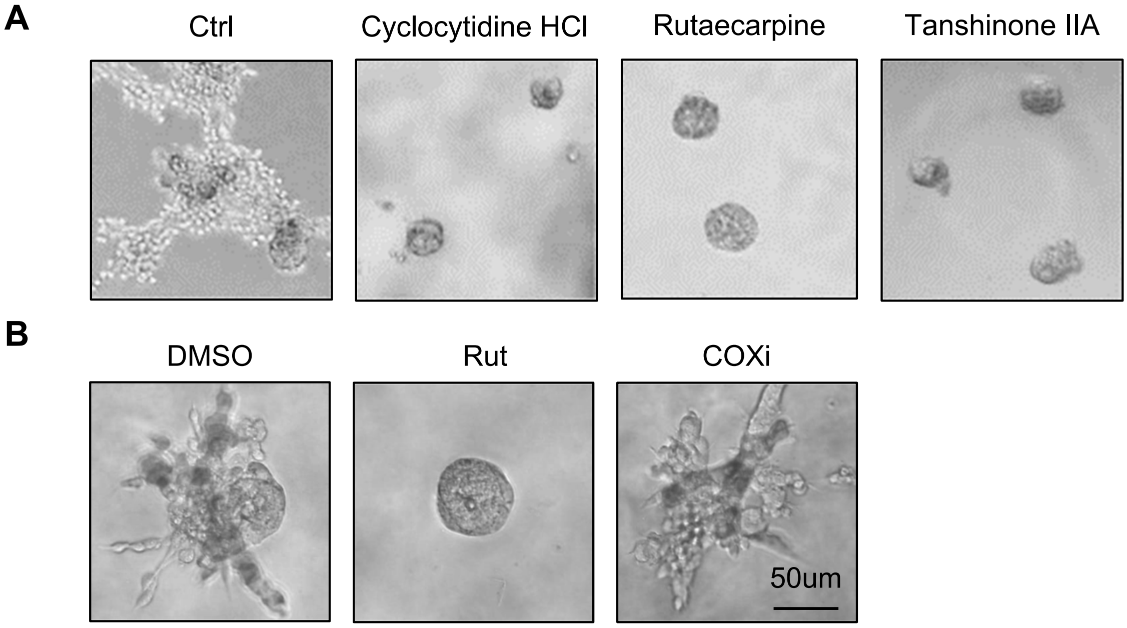

Extended Data Fig. 2 | Rutaecarpine has no effect in cell cycle, colony formation and migration in 2D cultured MDA-MB-231

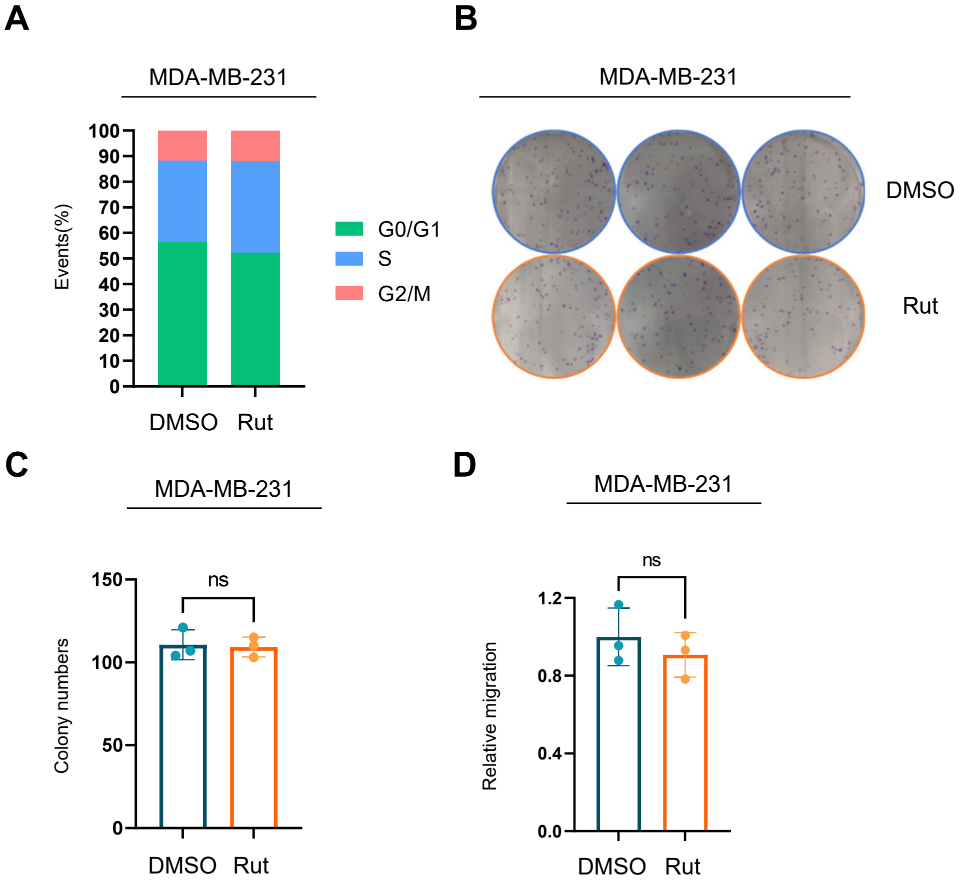

Extended Data Fig. 3 | FH but not CS is the direct target of Rutaecarpine

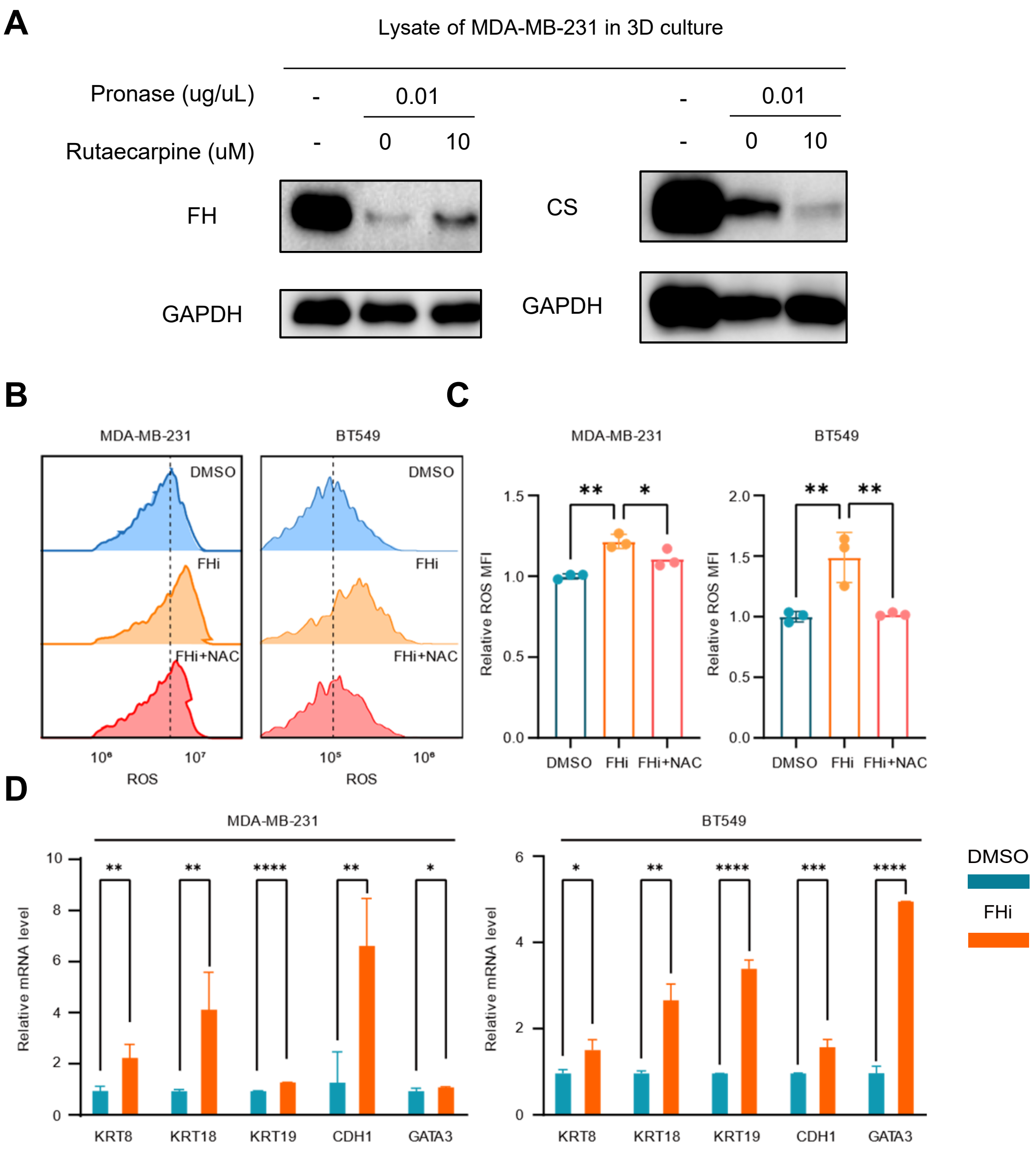

Supplement: Supplementary file 1 — Additional file 1: Figure S1. Related to Figs. 1 and 2: Rutaecarpine, but not COX2 inhibitor, induces differentiation of TNBC cells in 3D culture. A. Spheroid formation from MDA-MB-231 cells treated with the top three candidate drugs from the 3D screening, using DMSO as a vehicle control. Cyclocytidine HCl, 10 uM; Rutaecarpine, 10 uM; Tanshinone IIA, 10 uM. B. Spheroid formation from MDA-MB-231 cells treated with DMSO, rutaecarpine, and a COX2 inhibitor, rofecoxib. Figure S2. Related to Fig. 2: Rutaecarpine has no effect in either cell cycle, colony formation or migration of MDA-MB-231 cells. A. Cell cycle analysis of MDA-MB-231 cells purified from spheroids treated with DMSO or rutaecarpine in 3D culture. B. Colony formation assay of MDA-MB-231 cells treated with DMSO or rutaecarpine in 2D culture. C. Quantification of the colony formation assay of MDA-MB-231 cells. Unpaired Student’s t-test; ns, p > 0.05. D. Quantification of the migration assay of MDA-MB-231 cells with the treatment of DMSO or rutaecarpine in 2D culture. Unpaired Student’s t-test; ns, p > 0.05. Figure S3. Related to Fig. 5: FH, but not CS, is the direct target of rutaecarpine. A. DARTS assay to identified the target of rutaecarpine in spheroids formed by MDA-MB-231 cells using western-blotting. B. FCM test for the ROS of MDA-MB-231 and BT549 spheroids treated with DMSO, FH inhibitor, or a combination of FH inhibitor and NAC. C. Quantification of ROS MFI in spheroids formed by MDA-MB-231 and BT549 spheroids. One-way ANOVA; * p < 0.05; ** p < 0.01. D. qPCR of luminal marker genes in DMSO or FH inhibitor-treated MDA-MB-231 and BT549 spheroids. Unpaired Student’s t-test; * p < 0.05; ** p < 0.01; *** p < 0.001; **** p < 0.0001 [file 12967_2023_4396_MOESM1_ESM.pdf]
