# Supplementary material for: Rutaecarpine induces the differentiation of triple-negative breast cancer cells through inhibiting fumarate hydratase
Source: J Transl Med. 2023 Aug 18;21:553. doi: 10.1186/s12967-023-04396-w (PMC10436383; doi:10.1186/s12967-023-04396-w)
Supplement: Supplementary file 2 — Additional file 2: Primers for amplification of indicated genes. [file 12967_2023_4396_MOESM2_ESM.pdf]

Supplementary Table S1

**qRT-PCR primers used in this study**		
Gene name	Forward	Reverse
KRT8	CAGAAGTCCTACAAGGTGTCCA	CTCTGGTTGACCGTAACTGCG
KRT18	GGCATCCAGAACGAGAAGGAG	ATTGTCCACAGTATTTGCGAAGA
VIM	AGGCAAAGCAGGAGTCCACTGA	ATCTGGCGTTCCAGGGACTCAT
EPCAM	GCCAGTGTACTTCAGTTGGTGC	CCCTTCAGGTTTTGCTCTTCTCC
CDH1	GCCTCCTGAAAAGAGAGTGGAAG	TGGCAGTGTCTCTCCAAATCCG
GATA3	ACCACAACCACACTCTGGAGGA	TCGGTTTCTGGTCTGGATGCCT
KRT19	AGCTAGAGGTGAAGATCCGCGA	GCAGGACAATCCTGGAGTTCTC
ACTB	CACCATTGGCAATGAGCGGTTC	AGGTCTTTGCGGATGTCCACGT
